# Ontogenic stage-associated SA response contributes to leaf age-dependent resistance in *Arabidopsis* and cotton

**DOI:** 10.3389/fpls.2024.1398770

**Published:** 2024-07-29

**Authors:** Lanxi Hu, Jovana Mijatovic, Feng Kong, Brian Kvitko, Li Yang

**Affiliations:** Department of Plant Pathology, College of Agricultural and Environmental Sciences, University of Georgia, Athens, GA, United States

**Keywords:** temporal resistance, salicylic acid, ontogenic resistance, cotton, leaf maturation

## Abstract

**Introduction:**

As leaves grow, they transition from a low-microbe environment embedded in shoot apex to a more complex one exposed to phyllosphere microbiomes. Such change requires a coordinated reprogramming of cellular responses to biotic stresses. It remains unclear how plants shift from fast growth to robust resistance during organ development.

**Results:**

Here, we reported that salicylic acid (SA) accumulation and response were temporarily increased during leaf maturation in herbaceous annual *Arabidopsis*. Leaf primordia undergoing active cell division were insensitive to the elicitor-induced SA response. This age-dependent increase in SA response was not due to prolonged exposure to environmental microbes. Autoimmune mutants with elevated SA levels did not alter the temporal pattern dependent on ontogenic stage. Young *Arabidopsis* leaves were more susceptible than mature leaves to *Pseudomonas syringae* pv. *tomato* (*Pto*) DC3000 *cor−* infection. Finally, we showed a broadly similar pattern in cotton, a woody perennial, where young leaves with reduced SA signaling were preferentially invaded by a *Xanthomonas* pathogen after leaf surface infection.

**Discussion:**

Through this work, we provided insights in the SA-mediated ontogenic resistance in Arabidopsis and tomato.

## Introduction

Plant lateral organs start as small clusters of cells in the shoot apex and eventually grow into complex structures that are exposed to a diverse range of environmental microbes, including beneficial, commensal, and pathogenic microbes (Sinha, 1999; Bar and Ori, 2014). Young tissues are generally protected by surrounding pre-existing organs and therefore have limited exposure to microbes in their environment. During their development, these organs may shift from being susceptible to resistant to certain pathogens, which is referred to as ontogenic resistance or age-related resistance (ARR) (Develey-Riviere and Galiana, 2007; Berens et al., 2019; Hu and Yang, 2019). This phenomenon has been observed in various pathosystems (Bowling et al., 1994; Gusberti et al., 2013; Asalf et al., 2014; Twomey et al., 2015; Mansfeld et al., 2017). For instance, susceptibility of hop cones to powdery mildew gradually decreases after the transition from bloom to cone development (Twomey et al., 2015). Fruits of several cucurbit crops, including melon, butternut squash, watermelon, zucchini, squash, and pumpkin, also enhanced resistance to the oomycete pathogen *Phytophthora capsica*, as they increase in fruit size (Ando et al., 2009). Young cucumber (*Cucumis sativus*) fruits from some cultivars are highly susceptible to infection to the oomycete pathogen, *P. capsica*. However, they become resistant once they complete their exponential growth (Ando et al., 2009). Genes involved in multiple defense pathways, including physical barriers (e.g., cuticle thickness), penetration defense, flavonoid biosynthesis, oxidative stress, and microbe-associated molecular pattern (MAMP)- or effector-triggered immune responses, are enhanced in mature fruits (Ando et al., 2015). Comparing peel transcriptomes and metabolomes from mature “ARR-capable” and “ARR-defective” cucumber lines have linked an upregulation in terpenoid glycoside production during development to the ARR against *P. capsica* (Mansfeld et al., 2017). The regulatory mechanisms upstream of those age-related metabolisms and whether there are any signaling pathways conserved in operating ontogenic resistance are less clear.

Salicylic acid (SA) is a plant hormone vital in promoting resistance against biotrophic and hemibiotrophic pathogens (Huot et al., 2014; Berens et al., 2019; van Butselaar and Van den Ackerveken, 2020; Chan, 2022). The isochorismate synthase (ICS) pathway contributes to the pathogen-induced SA accumulation in *Arabidopsis* (Nawrath and Metraux, 1999). The AVRPPHB SUSCEPTIBLE 3 (PBS3) enzyme converts IC to SA by catalyzing the conjugation of IC and glutamate to produce isochorismate-9-glutamate (Rekhter et al., 2019). Then, the multidrug and toxin extrusion (MATE) transporter ENHANCED DISEASE SUSCEPTIBILITY 5 (EDS5) is responsible for transporting SA from chloroplast to cytosol. Mutations in EDS5, also known as *sid1*, lead to lower levels of SA upon pathogen infection (Nawrath and Metraux, 1999; Nawrath et al., 2002; Rekhter et al., 2019). In *Arabidopsis*, NONEXPRESSER OF PR GENES (NPR) family members act as major SA receptors in conducting SA-mediated defense signaling (Zhou et al., 2023). NPR1 and NPR3/4 can bind to SA with varying affinities, and a key conserved arginine residue in NPR1 (NPR1^R432^) and NPR3/4 (NPR3^R428^ and NPR4^R419^) is indispensable for SA binding (Fu et al., 2012; Ding et al., 2018; Liu et al., 2020). When associated with transcription factors, such as TGACG-binding (TGA) family members, NPR proteins act as either transcriptional co-activators (NPR1) or co-repressors (NPR3/4) to play opposing roles in regulating the transcription of downstream genes, including CAM-BINDING PROTEIN 60-LIKE G (CBP60g, AT5G26920) and SAR DEFICIENT 1 (SARD1, AT1G73805) (Fan and Dong, 2002; Despres et al., 2003; Ding et al., 2018). On the other hand, SA plays a pivotal role in plant development (Rivas-San Vicente and Plasencia, 2011; Li et al., 2022). SA modulates cell cycle transition in both positive and negative manners (Vanacker et al., 2001; Xia et al., 2009; Xu et al., 2017). SA also regulates specific developmental processes such as apical hook formation (Huang et al., 2020), flowering time (Martínez et al., 2004), root patterning (Pasternak et al., 2019), and leaf senescence (Morris et al., 2000).

Organ maturation has been associated with changes in disease resistance in several pathosystems, with varying hormone and metabolic pathways modulating these processes in coordination with ontogenic stages (Gusberti et al., 2013; Asalf et al., 2014; Twomey et al., 2015; Mansfeld et al., 2017). In *Arabidopsis*, a priority was given to either ABA-mediated abiotic or SA-mediated biotic stress responses at different leaf ages, enabling plants to balance abiotic and biotic stresses during growth (Berens et al., 2019). This suggests a temporal signaling coordination between fast growth and potent resistance during organ development. Still, much remains unknown about the amplitude and regulation of innate immunity during distinct developmental stages of an organ and if they are conserved across species. Our study revealed an ontogenic increase in SA accumulation and response during the maturation of *Arabidopsis* leaves. At the stage and the location of active cell division, leaves were compromised in activating SA responses and were associated with high susceptibility to pathogen. This age-dependent increase in SA response is not a result of prolonged exposure to environmental microbes. Furthermore, we found that auto-immune mutants with elevated SA did not alter the temporal pattern of SA response. Similar to the observations in *Arabidopsis*, we found that *Xanthomonas citri* pv. *malvacearum* displayed enhanced colonization in young cotton leaves. Likewise, SA signaling was reduced in young cotton leaves relative to expanded leaves. This implies a potential shared mechanism of ontogenic ARR between Brassicales and Malvales sister taxa.

## Results

### *Arabidopsis* mature leaves showed higher SA response than young leaves

**Figure 1:**
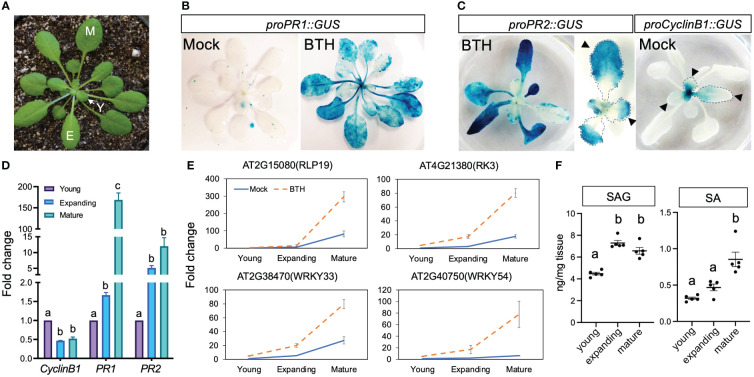
Mature leaves showed elevated SA accumulation and response. **(A)** A 5-week-old *Arabidopsis* plant grown in short-day condition. Y, young leaves; E, expanding leaves; M, mature leaves. Plants used in **Figure 1** were grown in soil. **(B)** The expression of *proPR1::GUS* at 24 h after mock or BTH treatment. Note the lack of induction in the center of BTH-treated plant where young (newly emerging) leaves were located (n>12 plants grown in soil were used for each treatment). **(C)** Complementary staining pattern between *proPR2::GUS* and *proCyclinB1::GUS*. The middle panel shows enlarged view of *proPR2::GUS* staining in young leaves. Note the staining first appeared at tip of leaves, which is complementary to the staining pattern of *proCyclinB1::GUS.* The lack of *proCyclinB1*::GUS and appearance of *proPR2::GUS* at the leaf tip is indicated by arrowheads (n>10 plants grown in soil were used for each treatment). **(D)** Expression patterns of *CyclinB1*, *PR1*, and *PR2* in young, expanding, and mature leaves. The fold change is relative to the expression of each gene in young leaves. Different letters above each date set indicate significant difference based on one-way ANOVA. Similar results were seen in two biological replicates. **(E)** Expression patterns of SA-responsive genes in young, expanding, and mature leaves. Leaves were harvested from 5-week-old plants grown in SD conditions. Error bars: standard error of three technical repeats. Similar results were observed in three independent biological replicates. Each biological replicate contained at least six leaves. Fold change is relative to the expression of each gene in mock-treated young leaves. **(F)** Accumulation of SA and SAG in young, expanding, and mature leaves. Young, expanding, and mature leaves were harvested from 5–7-week-old plants grown in SD conditions. Same plants were used to harvest young, expanding, and mature leaves. Different letters above each data set indicate significant difference based on one-way ANOVA.

*PATHOGENESIS-RELATED 1* and *2* (*PR1* and *PR2*) are canonical markers for SA-induced response (Cao et al., 1997; Nawrath and Metraux, 1999; Vogel and Somerville, 2000). In soil grown plants, we observed that the activity of β-glucuronidase (GUS) driven by the promoters of *PR1* and *PR2* were preferentially activated in mature leaves after treatment with benzothiadiazole (BTH), an SA analogue (**Figures 1A–C**), which is consistent with previously reported age-dependent accumulation of *PR1* transcripts (Berens et al., 2019). To better understand the cellular status of leaves at different ages, we examined the expression of *proCyclinB1::GUS*, a marker for actively dividing cells, in successive leaves and defined young, expanding, and mature leaves based on their staining pattern (**Figure 1C**). Leaves first mature at the distal end, indicated as the lack of *proCyclinB1*::GUS at leaf tip (arrowhead in **Figure 1C**), while *proPR2*::GUS often first appeared at the distal end of leaves (arrowhead in **Figure 1C**). We defined young leaves as those showing active cell division in the whole or a part of a leaf (>50% leaf area); expanding leaves have not reached their full size but with limited cell division; mature leaves reach full size without signs of senescence (**Figure 1A**). We noticed that *proPR2::*GUS was mostly evident in the tips of premature leaves, complementary to the staining pattern of *proCyclinB1*::GUS, indicating a suppression of SA response in actively dividing cells (**Figure 1C**). The ontogenic expression pattern was confirmed with qPCR-based quantification for transcript levels of these marker genes (**Figure 1D**). To further validate the observation, we tested the ontogenic stage expression of four additional SA-activated genes (*AT2G15080*, *AT4G21380*, *AT2G38470*, and *AT2G40750*) during leaf maturation (Yang et al., 2017) (**Supplementary Table S1**). Like *PR1* and *PR2*, these genes also showed high expressions in mature leaves after the BTH treatment (**Figure 1D, E**). Interestingly, despite the relatively low expression in mock-treated samples, the ontogenic increasing pattern remained, suggesting that endogenous SA accumulation or response was enhanced in mature leaves (**Figure 1E**). Indeed, mature leaves accumulated high level of free SA and SA metabolites, salicylic acid beta-glucoside (SAG), compared to young leaves or expanding leaves (**Figure 1F**). Taken together, our analysis suggested that both the SA accumulation and signaling pathway were temporally derepressed during leaf expansion.

### The ontogenic increase in SA response was not due to prolonged exposure to the phyllosphere microbiome

**Figure 2:**
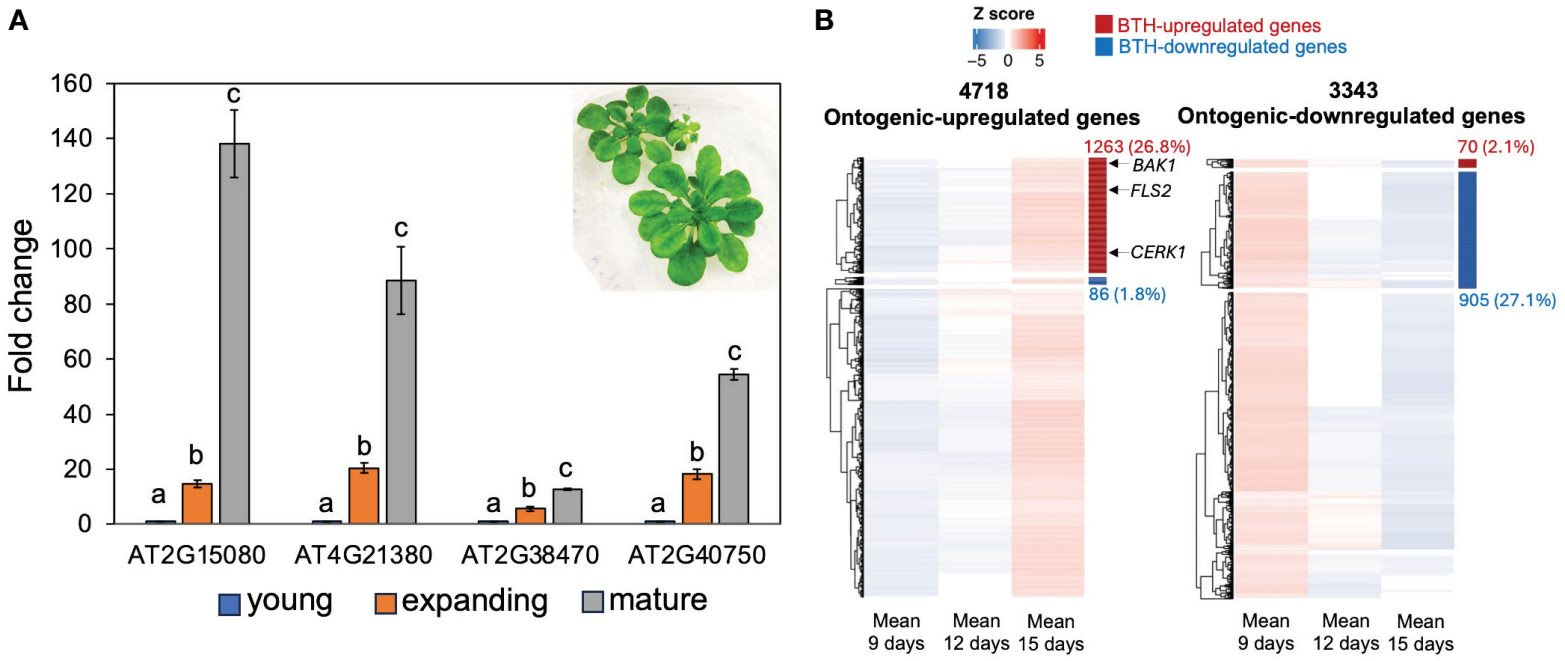
SA-responsive genes were upregulated during leaf expansion in sterile conditions. **(A)** The expression of SA-responsive genes in young, expanding, and mature leaves. Plants were grown in deep Petri dish (15 mm depth) and kept in an SD condition (see the image insert) for 3 weeks. The fold change is relative to the expression of each gene in young leaves. Error bars: standard error of three technical repeats. Different letters above each data set indicate significant difference based on one-way ANOVA. Similar results were observed from two independent experiments, each with 8–10 leaves at their respective ages. **(B)** Gene clusters show ontogenic stage expression pattern during leaf expansion. The left panel shows the increasing expression trajectory of 4,718 genes during leaf maturation; right panel shows the expression trajectory of 3,343 genes with decreasing patterns. These genes were detected in Pan et al. (2019) and combined in this work (see *Materials and methods*). SA-responsive genes were defined by Yang et al. (2017). Red and blue bars on the right side of the heatmap indicate BTH-activated and repressed genes, respectively. The heatmap position of *FLS2*, *BAK1*, and *CERK1* was indicated by arrows (see **Supplementary Table S2**).

To test if the ontogenic increase in SA response in mature leaves was due to prolonged exposure to the phyllosphere microbiome, we grew *Arabidopsis* seedlings under axenic short-day conditions and measured the basal expression levels of the forementioned four SA-responsive genes in young, expanding, and mature leaves harvested 4 weeks after planting (**Figure 2A**). All four genes showed higher expression in mature leaves, suggesting that ontogenic maturation of SA responses can occur independently of phyllosphere microbes. Nevertheless, we cannot exclude the possibility that prolonged exposure to environmental microbes may also contribute to the age-dependent enhancement of SA signaling. To obtain a global view of SA response during leaf expansion, we re-analyzed published transcriptomes of young, expanding, and mature leaves from plants grown in axenic conditions (Pan et al., 2019). A total of 4,718 and 3,343 genes showed ontogenic increases and decreases during leaf growth, respectively (Pan et al., 2019). Using SA-responsive genes defined by Yang et al. (2017), we found that 26.8% of ontogenic-increased genes were upregulated by SA (**Figure 2B**, **Supplementary Table S2**), including *CBP60g* and *SARD1*. Similarly, 27.1% of the temporally decreased genes were suppressed by SA (**Figure 2B**, **Supplementary Table S2**). In summary, SA-responsive genes counted for at least a quarter of genes that were temporally expressed during leaf maturation, suggesting an age-dependent tradeoff between growth and defense.

### *Arabidopsis* auto-immune mutants did not alter the temporal pattern of SA response

**Figure 3:**
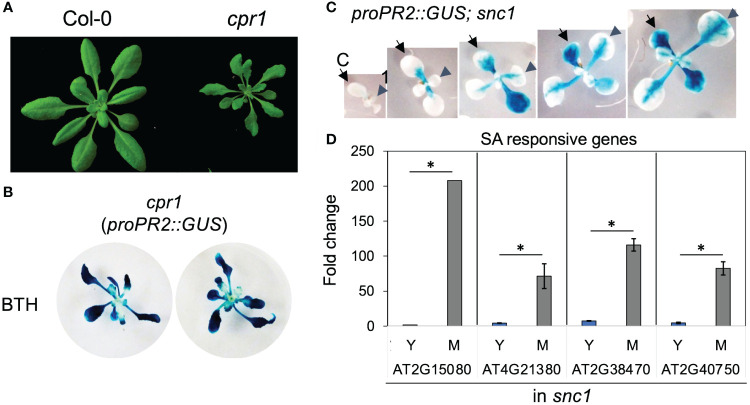
Young leaves had compromised SA response in autoimmune mutants cpr1 and snc1. **(A)** cpr1 mutant showing dwarf phenotype. **(B)** Staining of *proPR2::GUS* in *cpr1*. Note the reduced staining in the center of plants where young leaves were located (n>20 plants grown in soil for each genotype). **(C)** Staining of *proPR2::GUS* in *snc1* gain-of-function mutant. Note the gradual increase in staining during leaf (arrowhead) and cotyledon (arrow) expansion. C, cotyledon; 1, first leaf (n>20 plants grown in soil for each genotype). **(D)** The expression of SA-responsive genes in young and mature leaves from *snc1* mutant. Error bars: standard error of three technical repeats. Similar results were observed in two independent biological replicates. *Significant difference between young and mature leaves using Student’s t-test. Y, young leaves; M, mature leaves. Similar results were obtained from two independent experiments. Fold change is relative to the expression of each gene in young leaves.

Auto-immune mutants have enhanced disease resistance due to hyperactivation of a branch of plant innate immunity, including the SA signaling (van Wersch et al., 2016). To investigate if the temporal pattern of SA response could be maintained in auto-immune mutants, we measured the activity of the *PR2* promoter in gain of function *SUPPRESSOR OF NPR1–1, CONSTITUTIVE 1* (*snc1*), and loss-of-function *CONSTITUTIVE EXPRESSER OF PR GENES 1* (*cpr1*) mutants (Bowling et al., 1994; Li et al., 2001). Both mutants accumulate a higher level of SA than Col-0 wild type and are more resistant to biotrophic pathogens (Bowling et al., 1994; Li et al., 2001). As previously reported, *cpr1* mutant was smaller than Col-0 (**Figure 3A**). Notably, we found that although these mutants exhibited intense *proPR2::GUS* staining, their young leaves were still compromised in *PR2* promoter activity, indicating that the SA response was constrained in auto-immune young leaves (**Figures 3B, C**). Consistent with the staining pattern of *proPR2::GUS*, expressions of SA-responsive genes showed temporal increases from young to mature leaves in the *snc1* mutant background (**Figure 3D**). Thus, the hyperactivation of SA response in autoimmune mutants was not due to a precocious activation in young leaves.

### Premature *Arabidopsis* leaves were more susceptible to *Pseudomonas syringae* pv. tomato DC3000 coronatine−

**Figure 4:**
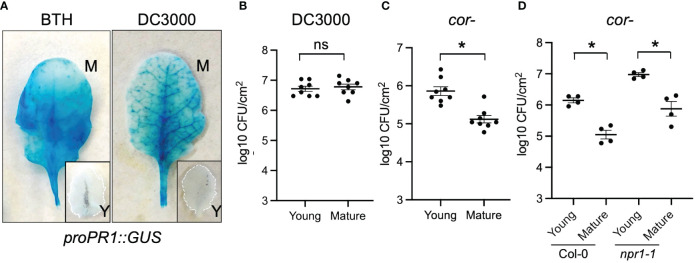
Young *Arabidopsis* leaves were compromised in defense against *Pto* DC3000 *cor−* stain. **(A)** *Pto* DC3000 and BTH triggered *proPR1::GUS* expressions in mature (M) but not young (Y) leaves. Leaves were sampled 24 h after BTH or *Pto* DC3000 infiltration. **(B)** Mature and young leaves showed the same level of susceptibility to *Pto* DC3000. Samples were collected at 48 h after *Pto* DC3000 infiltration (n=8). **(C)** Young leaves were more susceptible to *Pto* DC3000 *cor−* than mature leaves. Samples were collected at 48 h after *Pto* DC3000 *cor−* infiltration (n=8). **(D)** The difference in susceptibility between young and mature leaves were maintained in *npr1–1* mutant. Samples were collected at 48 h after *Pto* DC3000 infiltration. From panels **(B–D)** ns, no significance. *Significant difference between young and mature leaves using Student’s t-test. Similar results were obtained from at least three independent experiments.

Next, we tested whether young and mature leaves differentially activate SA response during pathogen infection. We challenged young and mature *Arabidopsis* leaves with *Pseudomonas syringae* pv. *tomato* (*Pto)* DC3000, a hemibiotrophic bacterial pathogen. As their responses to BTH, mature leaves showed a strong response to *Pto* DC3000 infection indicated by a high activation of *proPR1::GUS* (**Figure 4A**). In contrast, the amplitude of activation was compromised in young leaves (**Figure 4A**). This suggests that the pathogen-triggered SA signaling was temporally elevated during leaf ontogenesis.

We further assessed whether the ontogenic maturation of SA response contributes to disease resistance by challenging premature and mature leaves with *Pto* DC3000 and *Pto* DC3000 *cor−* (coronatine defective) strains. Coronatine, a phytotoxin produced by *Pto* DC3000, dampens *Arabidopsis* SA-mediated immunity by activating jasmonic acid responses (Zheng et al., 2012). Consistent with the previous report, *Pto* DC3000 multiplication was not affected by leaf age (**Figure 4B**) (Berens et al., 2019). We reasoned that the presence of coronatine in wild-type *Pto* DC3000 may help it counteract the enhancement of the SA pathway from young to mature leaves (Zheng et al., 2012). In line with this, we found that mature leaves were more resistant than young leaves to the *Pto* DC3000 *cor−* strain, implying that the ontogenic resistance in mature leaves can be linked to the enhanced SA-mediated defense (**Figure 4C**). Surprisingly, blocking SA signaling in *npr1* mutant did not abolish the difference of bacterial multiplication between young and mature leaves (**Figure 4D**), suggesting NPR1-independent pathways contribute to ontogenic resistance. In support of this, the expressions of pattern-triggered immunity (PTI) components including *FLAGELLIN SENSING2* (*FLS2*, *AT5G46330*), *BRI1 ASSOCIATED RECEPTOR KINASE 1* (*BAK1, AT4G33430)*, and *CHITIN ELICITOR RECEPTOR KINASE 1* (*CERK1, AT3G21630)* were higher in mature leaves than in young leaves (**Figure 2B**, **Supplementary Table S2**), with the expression pattern of *FLS2* being consistent with what was reported previously (Zou et al., 2018).

### Young cotton leaves were more susceptible to *Xanthomonas citri* pv. *malvacearum* infection than mature leaves

**Figure 5:**
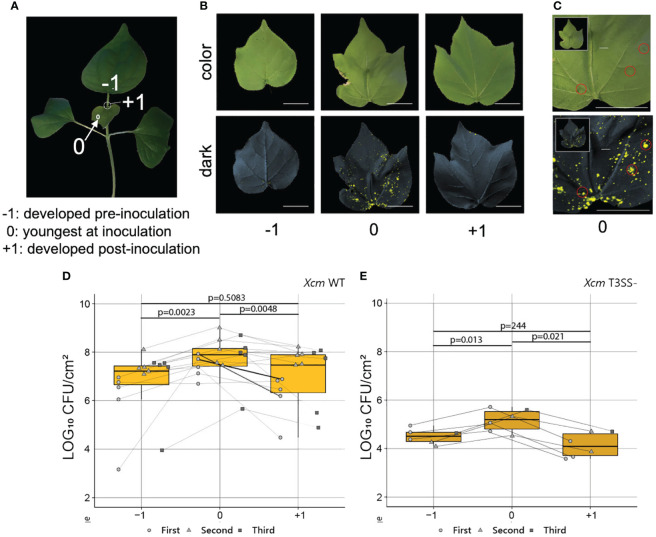
Young cotton leaves were preferentially colonized by *Xcm*. **(A)** A 2-week-old cotton plant. “−1”, mature leaves developed pre inoculation; “0”, young leaves actively developing at the time of inoculation; “+1”, young leaves actively developing post-inoculation. **(B)** Representative color and 2-min exposure auto-bioluminescence overlay images of infected cotton true leaves developed pre and post *Xcm* 4.02 WT Tn7LUX dip inoculation. **(C)** Correlation of symptoms developed 2 weeks post-Xcm 4.02 WT Tn7LUX dip inoculation and the zones of auto-bioluminescence. **(D, E)** Bacterial populations in log10 CFU/cm^2^ value post-*Xcm* 4.02 WT Tn7LUX **(D)** and Xcm 4.02 ΔhrcV Tn7LUX **(E)** infection, combined from three experimental replicates. Boxplots show mean ± SD. p-values were calculated using Student’s t-test.

To test whether the findings in herbaceous *Arabidopsis* could be generalized in distantly related species such as cotton, we dip inoculated 2-week-old cotton seedlings with at least one fully expanded true leaves with *Xcm* 4.02 wild-type Tn*7*LUX or *Xcm* 4.02 Δ*hrcV* Tn*7*LUX (T3SS-, defective in Type III secretion systems) (Mijatović et al., 2021). To track the pattern of *Xcm* colonization, we recorded bacterial auto-bioluminescence and examined bacterial population in leaves at different levels of maturity (**Figure 5**). As it takes up to 3 weeks for disease development in cotton, we marked the youngest actively expanding leaf (leaf “0”) at the time of inoculation to monitor relative leaf age (**Figure 5A**). Image overlay of auto-bioluminescence patterns revealed variable colonization dependent on leaf age at the time of inoculation (**Figure 5B**). As observed in our previous work (Mijatović et al., 2021), both *Xcm* 4.02 WT Tn*7*LUX (**Figures 5C, D**) and *Xcm* 4.02 Δ*hrcV* Tn*7*LUX (**Figure 5E**) can colonize cotton tissue, but at different bacterial loads. The youngest actively developing leaves (leaf “0”) at the time of inoculation consistently showed increased Xcm auto-bioluminescence when compared to leaves developed pre-noculation (leaf “−1”) and post-inoculation (leaf “+1”) (**Figure 5B**). It is worth noting that auto-bioluminescence observed from overlayed images corresponds with the location of water-soaking disease symptoms in *Xcm* 4.02 WT Tn*7*LUX inoculated seedlings (**Figure 5C**). Comparison of *Xcm* loads in tested leaf samples revealed differences in bacterial population of both the WT and *hrcV* mutant between leaves of different age (**Figures 5D, E**), consistent with the ontogenic enhancement in basal resistance observed in *Arabidopsis*. Both pathogens consistently displayed higher colonization in the youngest actively developing leaves (leaf 0) at the time of inoculation (**Figures 5C, D**). Lower bacterial populations in leaves developed pre-inoculation (leaf “−1”) indicate that more mature cotton leaves have enhanced defense responses to *Xcm* infection. This is in line with the compromised basal defense response observed in young leaves of *Arabidopsis*.

### SA and SAG accumulation varied in young and mature cotton leaves

**Figure 6:**
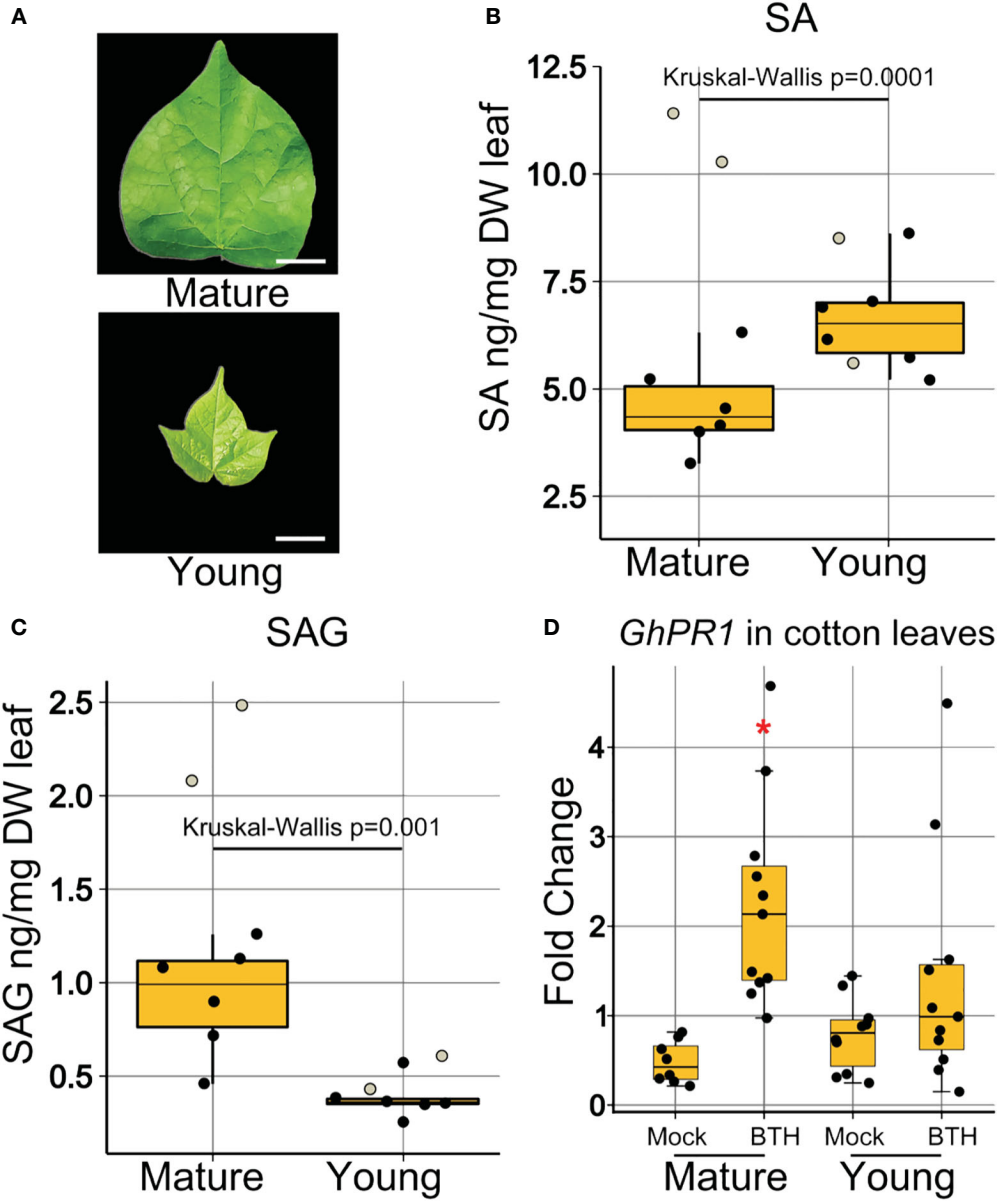
Young cotton leaves were compromised in SA signaling. **(A)** Visual representation of the morphology of collected mature and young leaf samples. Bars represent 1 cm length. **(B)** Concentration of free SA in ng/mg of dry leaf weight. **(C)** Concentration of SAG in ng/mg of dry leaf weight. Boxplots show mean ± SD. Eight samples of five pooled biological replicates each represented as jitter dots on boxplots. Two dots representing two samples (white filled) were not used in statistical analysis, as they were found to be outliers outside of the first and third quartile. Data were analyzed by Kruskal–Wallis test because the results met non-parametric requirements. **(D)** Expression of SA-responsive genes in mature and young cotton leaves at 24-h post-BTH treatment. Points represent biological replicates from three experimental replicates. Boxplots show mean ± SD. Differences in gene expression from 3 experimental replicates were pooled and analyzed using ANOVA. *Significant difference between *Gh*PR1 expression in young and mature leaves.

Having determined that young leaves at the time of inoculation are more heavily colonized, we hypothesized that, similar to *Arabidopsis*, ontogenic differences in SA accumulation or SA signaling might mediate age-related susceptibility to *Xcm* in cotton. Samples representing mature and young cotton leaves (**Figure 6A**) were collected, lyophilized, macerated, and examined by quadrupole time-of-flight mass spectrometry (QTOF-MS). Analysis of accumulation of free SA revealed higher concentration in young leaves than in mature leaves (**Figure 6B**). Conversely, the inactive vacuole stored SAG was at a higher concentration in mature leaves compared to young leaves (**Figure 6C**). These results indicate that the cause of young leaf age-related immunodeficiency in cotton is likely not linked to the free SA level but may be correlated with accumulations of SAG or impaired SA signaling.

### *GhPR1* induction by BTH differs between young and mature cotton leaves

To further elucidate the contribution of SA to the immunodeficiency in young cotton leaves, we investigated the accumulation of *GhPR1*, an SA-responsive gene in cotton and an orthologue of *Arabidopsis PR1*. We dip inoculated cotton leaves with BTH and recorded expression of *GhPR1* at 24 h after treatment in mature and young (**Figure 6D**) leaves. *GhPR1* was induced by BTH treatment in mature leaves at 24 h post-inoculation, but induction in young leaves was not statistically significant (**Figure 6D**). Thus, the SA signaling response was elevated in mature leaves in cotton, which correlated with the enhanced disease resistance against *Xcm* 4.02 strains (**Figures 5D, E**).

## Discussion

Leaf development involves two types of cell divisions. In the first type, cells undergo rapid proliferation with a high incidence in the initial primordium. When these cells exit the mitotic cycle, they continuously expand in volume and increase further in size. Endoreduplication is a variant of the cell cycle in which cells undergo DNA replication without subsequent mitosis. During plant organ ontogenesis, cell division often first occurs densely at the distal half of an organ and then progresses toward the base over time. This common spatial pattern is known as the basipetal gradient (Nath et al., 2003; Ori et al., 2007; Nelissen et al., 2012; Du et al., 2018). Resemble to this gradient of cell proliferation, in our work, we observed that SA response indicated by *PR1* and *PR2* promoter activities first appeared in the distal part of an expanding leaf where cells mature earlier than those in the proximal end of a leaf (**Figure 1**), suggesting that the elevated SA response is associated with cellular maturation status in a leaf. On the other hand, genes involved in regulating endoreduplication could play dual roles in plant immunity. For example, SIAMESE (SIM) and SIAMESE RELATED 1 (SMR1) act synergistically to promote endoreplication (Churchman et al., 2006; Hamdoun et al., 2016). Cells in a *sim smr1* double mutant showed a low endoreduplication index. Interestingly, *smr1* mutant also partially suppressed the dwarfism and cell death phenotypes caused by high SA levels in *acd6–1*, indicating that SMR1 may be a positive regulator of plant defense (Hamdoun et al., 2016). These studies indicate that SMR1 may coordinate the increase in ploidy level and SA response during leaf maturation. On the other side, overexpressing a D-type CYCLIN 3;1 (CYCD3;1) restrains endoreduplication (Menges et al., 2006). *Cycd3;1,2,3* mutants showed a higher ploidy level than wild type and were more susceptible to a virulent strain *Pseudomonas syringae* pv. *maculicola ES4326*, which could be rescued by BTH (Hamdoun et al., 2016). In another case, OMISSION OF SECOND DIVISION (OSD1) and UV-B-INSENSITIVE 4 (UVI4) inhibited endoreduplication because their loss-of-function mutants showed high endoreduplication indices (Bao and Hua, 2014). However, overexpressing OSD1 and UVI4 can enhance immunity to a bacterial pathogen (Bao and Hua, 2014). Thus, the level of endoreduplication is not always positively correlated with high SA response or elevated defense, indicating a more complex crosstalk.

Despite cell division, other developmental constraints may contribute to SA-mediated ontogenic resistance. Key steps of SA biosynthesis include the synthesis of isochorismate that are processed in chloroplasts and then transported to cytoplasm by EDS5, a MATE family transporter (Serrano et al., 2013; Rekhter et al., 2019; Lefevere et al., 2020). Chloroplast differentiation is tightly associated with leaf maturation, from proplastids in cells of primordia to chloroplasts in mature leaves. During chloroplast biogenesis, thylakoids are formed, and proteins required for photosynthesis are arranged within the thylakoid (Jarvis and López-Juez, 2013; Gugel and Soll, 2017; Cackett et al., 2022). Premature leaves contain a reduced number of thylakoids per chloroplast (Gugel and Soll, 2017). The transition from plastids to mature chloroplasts during leaf development is shared between monocots and dicots seedlings (Pogson et al., 2015), although it is not clear if fully differentiated chloroplasts are more potent in producing SA precursors. Still, reduced SA biogenesis alone is not sufficient to explain the diminished SA response because young leaves were also compromised in SA signaling transduction upon SA treatment or pathogen infection (**Figures 2**, **3**, **6**).

In our research, both SA biogenesis and signaling were developmentally regulated. In *Arabidopsis*, SA is glucosylated to SAG or SA glucose ester (SGE) (George Thompson et al., 2017). SAG is mostly accumulated in the vacuole, and the majority of SGE is located in the cytosol (Vaca et al., 2017). It is proposed that in *Arabidopsis*, SAG acts as a long-term storage form of SA, while SGE, due to its ease of modification for active SA release, acts as a mobile SA factor (Vaca et al., 2017). Our results indicate that, unlike *Arabidopsis*, free SA is more abundant in young cotton leaves than that in mature leaves (**Figure 6B**), while the SAG concentration was consistently higher in mature than in young leaves (**Figures 1E**, **6C**). It is possible that free SA and SAG played distinct roles in ontogenic resistance of *Arabidopsis* and cotton leaves, which remain to be investigated. We also observed a significant induction of *GhPR1* in mature but not young cotton leaves at 24 h post-BTH treatment. These data were further supported by the disease resistance output.

Like many *Xanthomonas* pathogens, *Xcm* infection of cotton takes place over the course of weeks rather than over the course days as seen for the *Pto* DC3000-*Arabidopsis* interaction. However, a similar pattern of ontogenic resistance was observed in both pathosystems. We observed increased *Xcm* colonization of young cotton leaves actively developing at the time of inoculation based on both bacterial load and auto-bioluminescence localization pattern. Similar age-dependent reductions in bacterial loads were observed for both the WT and disarmed *Xcm hrcV* T3SS mutant, which is consistent with decreased basal immunity in young cotton leaves. Although cotton is grown as an annual crop, it is a perennial plant that develops a woody stem. These observations suggest an SA-dependent ontogenic resistance pathway exists in both herbaceous annual and woody perennial plants, despite the differences in infection strategies and disease progression.

It is interesting that the mutation in *NPR1* did not fully abolish the ontogenic resistance as we observed in *Arabidopsis*. Other genetic factors in charge of ontogenic resistance may be tied to genes involved in the downstream function of different NPR proteins, such as key transcription factors in SA signaling: TGAs and WRKYs. In addition to genes involved in SA pathway, we also noticed that key components of the PTI signaling in *Arabidopsis* were upregulated during leaf maturation (**Figure 2B**, **Supplementary Table S2**). The activation of either SA or PTI responses can result in stunted growth, spontaneous cell death, and other developmental defects. These negative impacts on plant growth could be more detrimental in young tissues than in old tissues. Thus, the age-dependent activating of PTI or SA responses may have evolved to protect young tissues from strong immune damages.

Overall, we showed that age-dependent SA signaling, and part of the SA accumulation, are positively associated with the enhanced disease resistance in both mature *Arabidopsis* and cotton leaves. This positions SA as a potentially conserved pathway regulating ontogenic resistance across woody and herbaceous species. As cotton displays both apical and axillary growth throughout the growing season, young cotton leaves are continuously available as a potential entry point for pathogen invasion. Given the diversity of SA biogenesis and signaling mechanisms (Ullah et al., 2023), further investigations of SA molecular components in cotton and other plants could shed lights on the conservation and divergence of SA-mediated ontogenic resistances.

## Materials and methods

### Plant material, growth conditions, and bacterial strains

For *Arabidopsis*, wild type, transgenic lines, and mutants utilized in this study were in a Columbia-0 (Col-0) genetic background. The plants were sown on Fafard #3 Mix propagation soil, and the planted seeds were placed under 4°C for 2 days before transferring to a growth room with a temperature of 22°C and 45% humidity. A short-day condition with a photoperiod of 9 h light and 15 h dark and 180 μmol m^−2^ s^−1^ was employed, and the lighting was provided by a 5:3 combination of white (USHIO F32T8/741) and red-enriched (interlectric F32/T8/WS Gro-Lite) fluorescent lights. Plant age was counted from the day when seeds were transferred to the growth room. Young, expanding, and mature leaves were collected from 5–7-week-old plants, using the same plants for each leaf age. These leaves were in adult stage beyond leaf 10. Under axenic conditions, plants grown on autoclaved 1/2 MS media had leaves harvested from 3-week-old plants in the same manner as those grown in soil.

For cotton, seeds of an Xcm-susceptible cotton cultivar Deltapine 1747NR B2XF (DP 1747NR B2XF) were sown (one to two seeds/pot) directly in SunGrow Professional growing potting mix (Farfard 3B, Sungro; Agawam, MA) in 9-cm pots. Pots were incubated in a growth chamber (Conviron A1000) with a 12-h day at 26°C and a 12-h night at 23°C.

*P. syringae* DC3000 wild type and DC3000 *cor−* mutant strains were described in (Yang et al., 2017). *Xanthomonas* 4.02 WT and Δ*hrcV* mutant creation and Lux Tn7 tagging were described previously (Mijatović et al., 2021).

### Bacterial infection

*Arabidopsis* infection with *P. syringae.* Bacteria strains were cultivated on King’s B solid medium [10 g/L peptone, 10 g/L glycerol, 15 g/L agar, 10 g/L Tryptone, 10 mL 10% K_2_HPO_4_ (10 g K_2_HPO_4_/100 mL), and 10 mL 10% MgSO_4_ (10 g MgSO_4_ anhydrous/100 mL)] at 28°C. The medium was supplemented with rifamycin for selection, and cycloheximide was added to prevent fungal growth. Before inoculation, bacterial stocks were streaked on a plate and allowed to grow for 2 days before being re-streaked one day prior to hand inoculation. To initiate infiltration, bacteria were collected from the plate and suspended in a 10-mM MgCl_2_ solution. Bacteria were inoculated into *Arabidopsis* leaves using a needless syringe. The initial inoculum was at OD = 0.1 and with 500 times dilution. After inoculation, plants were covered with transparent lids for 1 h. Each sample was composed of four-leaf disks obtained from four individual leaves using a corer. Two days after infiltration, leaf samples were collected and homogenized using an OMNI International homogenizer, diluted serially, and plated on KB plates with 10 µL of bacterial suspension per sample. The plates were incubated in a 28°C incubator for 2 days, and colony forming units were counted manually and normalized based on the inside area of the corer.

Cotton infection with *Xanthomonas citri* pv. *malvacearum*. Two-week-old cotton seedlings of the Xcm-susceptible (DP 1747NR B2XF) cotton cultivar, with the first two true leaves developed, were dip inoculated by submerging in 400 mL of bacterial cell suspension for 30 s. Inoculum (∼5 × 10^8^ CFU/mL) was made by suspending plate-cultured cells cultured 48 h on LB agar augmented with 50 µg/mL Kanamycin of *Xcm* 4.02 WT Tn*7*LUX or *Xcm* 4.02 Δ*hrcV* Tn*7*LUX in Milli-Q H_2_O with 0.02% Silwet adjuvant (Silwet L-77 Ag, PhytoTech). The youngest actively developing leaf at the time of inoculation was marked with plastic flagging for future analysis. This experiment was repeated three times. The Student’s t-test was conducted to analyze *Xcm* load using R studio.

Leaves that developed from inoculated cotton seeds were collected and imaged at 2 weeks after transplanting into soil. For each sample, three images were taken with the following conditions: color (Samsung galaxy s10+), grayscale (70% aperture) (Analytic Jena UVP ChemStudio, Upland, CA), and no light with 2 min of exposure time (100% aperture) (Analytic Jena UVP ChemStudio, Upland, CA). Grayscale and 2-min exposure images were overlayed in Adobe Photoshop CC 2020. Details regarding image processing using Adobe Photoshop (2022) are as previously described (Mijatović et al., 2021).

Bacterial load quantification (CFU/cm^2^) was done by collecting 4 × 4 mm^2^ leaf disks with a biopsy punch, suspending it in 200 µL water in 2 mL, screw-cap with o-ring plastic tubes (Fisher Scientific) with three high-density 3 mm zirconium beads (Glen Mills). Maceration was done twice for 1 min each at 1,750 Hz, using a GenoGrinder (SPEX SamplePrep, Metuchen, NJ). The macerate was then 10-fold serially diluted and plated on LB agar media supplemented with 50 μg/mL of kanamycin and 80 μg/mL cephalexin. Bacterial populations were determined after incubation for 2 days at 28°C.

### HPLC-QTOF-MS

For *Arabidopsis*, adult Col-0 leaves harvested from 5-, 6-, or 7-week-old plants grown in soil constituted one biological replicate for young (eight leaves per replicate), expanding (eight leaves per replicate), and fully mature leaves (three leaves per replicate). Five biological replicates were prepared for each developmental stage. Each biological replicate was flash frozen in a 50-mL falcon tube. The leaves were lyophilized and proceed for the high-performance liquid chromatography coupled with quadrupole time-of-flight mass spectrometry (HPLC-QTOF-MS) following the method described in Hu et al (Hu et al., 2023). The amount of salicylic acid beta-glucoside (SAG) and salicylic acid (SA) were quantified in the unit of nanogram metabolite per milligram of tissue (ng/mg tissue).

For cotton, eight samples, each consisting of five pooled biological replicates were flash frozen in 50-mL conical tubes and lyophilized for 24 h at 0.008 mbar and −84°C in a freeze dryer (Labconco). Following lyophilization, samples were macerated in 50 mL conical tubes with three 3-mm steel beads, two times for 2 min at 1,750 Hz, using a GenoGrinder (SPEX SamplePrep, Metuchen, NJ). Samples were then sent for metabolite extraction and QTOF analysis at Warnell School of Forestry and Natural Resources, UGA. The amount of SAG and SA were measured and calculated in the unit of nanogram metabolite per milligram of dry weight (ng/mg DW).

### qRT-PCR

For *Arabidopsis*, RNA extraction was conducted using an Omega biotek EZNA plant RNA kit (Omega Biotek) or a RNeasy Plant Mini Kit (Qiagen), and quantitative PCR (qPCR) was performed on an Applied Biosystems QuantStudio 1 Real-Time PCR system with SYBR Green master mix (Applied Biosystems). The following PCR conditions were used: 95°C for 5 min, followed by 40 cycles of 95°C for 15 s, 56°C for 30 s, and 72°C for 20–30 s. Reference gene *SAND* (*AT2G28390*) was utilized. *SAND* expression was not affected by leaf age (Klepikova et al., 2016) (Pan et al., 2019) or infection (Yang et al., 2017). Relative expression was determined using the relative standard curve method, while delta–delta CT was used when the PCR efficiency for the primers was previously established to be at least 95%. The oligonucleotides used in this study are listed in **Supplementary Table S1**.

For cotton, RNA was extracted following the woody plant protocol from Gambino et al. (2008). Briefly, 900 µl of extraction buffer (2% CTAB, 2.5% PVP-40, 2M NaCl, 100 mM Tris–HCl pH 8.0 and 2% β-mercaptoethanol added just before use) preheated at 65°C were added to ~150 mg of cotton tissue sampled previously, ground in liquid nitrogen with three 3-mm zirconium beads (Glen Mills) and one metal bead, macerated two times for 1 min at 1,750 Hz, using a GenoGrinder (SPEX SamplePrep, Metuchen, NJ). The tubes were then vortexed and incubated at 65°C for 10 min. An equal volume of chloroform:isoamyl alcohol (24:1 v/v) was added. Tubes were then inverted vigorously and centrifuged in a pre-cooled centrifuge for 10 min at 4°C, 11,000 RCF. Following centrifugation, the supernatant was extracted into clean 1.5-mL tubes and a second chloroform:isoamyl alcohol extraction was done. The supernatant was then transferred into a new microcentrifuge tube with 400 µL 3M LiCl. The mixture was then incubated on ice for 30 min and incubated at −20°C overnight. The next day, the RNA was pelleted by centrifugation at 21,000 RCF for 20 min at 4°C. The supernatant was decanted, and the pellet was resuspended in 500 µL of SSTE buffer consisting of 10 mM Tris–HCl pH 8.0, 1 mM EDTA pH 8.0, 1% SDS, and 1M NaCl preheated at 65°C. An equal volume of chloroform:isoamyl alcohol was added, and the mixture was centrifuged for 10 min at 4°C, 11,000 RCF. The supernatant was then precipitated with 0.7 volumes of cold isopropanol and centrifuged for 15 min at 4°C, 21,000 RCF. The RNA pellet was subsequently washed with 70% EtOH in nuclease-free water, dried, and resuspended in nuclease-fee water.

Cotton RNA samples were subjected to an off-column DNase treatment using a TURBO DNA-free kit (Thermo Fisher Scientific) following the manufacturer’s recommendations. Following DNase treatment, the samples were cleaned using New England Biolabs (NEB) Monarch RNA Clean and Concentrate kit, following manufacturer’s instructions. The cDNA library was then created using qScript cDNA supermix (Quantabio) according to manufacturer’s instructions. All RNA and cDNA samples were tested for genomic DNA (gDNA) contamination before qPCR analysis using cotton *GhGAPDH* (*glyceraldehyde 3-phosphate dehydrogenase*) gene primers (**Supplementary Table S1**). For the qPCR reaction, 1–5 ng of cDNA template was used (standardized to the same concentration per experimental replicate). Conditions of the qPCR were kept identical throughout all runs within experimental replicates following the protocol of Smith et al. (2018). Amplification of cDNA was done in 10 µl reactions using Luna Universal qPCR Master Mix (NEB), 0.25 µM primers, and 2 µL of standardized cDNA. Master mixes and primers were pre-aliquoted for single use and stored at −20°C. All PCR reactions were run in triplicate wells, and sample-well organization was kept identical between plates within experimental replicates. We followed the default thermal cycling protocol in the StepOne software v2.3 (Thermo Fisher Scientific) with real-time capture of SYBR green and ROX (passive reference) fluorescence as follows: 10 min at 90°C, followed by 40 cycles of 95°C for 15 s and 60°C for 1 min, with camera capture at the end of each cycle. A melt curve was generated after the 40th cycle, using the following parameters: 95°C for 15 s, 60°C for 1 min, then a slow ramp (0.3°C/s) to 95°C, with camera capture. All runs were conducted on the Step One Plus real-time PCR system (Thermo Fisher Scientific). For housekeeping gene controls, we used previously published *GhUBQ1* (Cox et al., 2017) and *GhGAPDH* (McGarry et al., 2016) primers (**Supplementary Table S1**). Relative quantification of *GhPR1* was calculated as described by Smith et al. (2018) following the modified Pfaffl method (Hellemans et al., 2007; Pfaffl, 2007). Three biological and three technical replicates were included in each run. Differences in gene expression were determined by comparing all 24-h data to 6-h mock-treated sample set. Relative expression was calculated using average Cqs and PCR efficiencies. The E^ΔCq values from reference genes (*GhGAPDH*, *GhUBQ1*) (**Supplementary Table S1**) were geometrically averaged to make the Normalizing Factor (NF). NF is divided into the E^ΔCq of *GhPR1* to give Fold Change (FC). The FC of all three experimental replicates was analyzed using Rstudio to determine significant difference based on one-way ANOVA.

### GUS staining assay

Plants containing *proPR2::GUS*, *proPR1::GUS*, or *proCyclinB1::GUS* were harvested at the specified time after planting. To prepare the GUS solution, a mixture of 0.1 M NaPO_4_ (pH 7.0), 10 mM EDTA, 0.1% Triton X-100, 1 mM K_3_Fe(CN)_6_, and 2 mM X-Gluc (dissolved in N,N-DMF and freshly made) was vacuum infiltrated into the plants. Samples were incubated at 37°C for various time from 1 h to 24 h. The staining solution was then substituted with 70% ethanol. The tissues were washed multiple times with 70% ethanol until the chlorophyll in the leaves was removed. The GUS-stained leaves were then observed under a dissecting microscope (VWR).

### Re-analysis of published RNA-seq datasets

We reanalyzed cluster 1 (3,918 genes), cluster 2 (800 genes), cluster 3 (1,512 genes), and cluster 4 (1,831 genes) derived from Pan et al. (2019). Clusters 1 and 2 were combined as 4,718 upregulated genes during leaf development. Clusters 3 and 4 were combined as 3,343 downregulated genes during leaf development. In Excel, we calculated the arithmetic mean and standard deviation of transcripts per million (TPM) for each gene using three replicates derived from 9 days, 12 days, and 15 days, respectively. The Z-score for each gene, replicate, and day was calculated with the following formula: (observed TPM − arithmetic mean)/standard deviation. The mean Z-score per gene per day was generated by averaging the Z-score of the three replicates. Next, we overlapped the BTH-responsive genes identified by Yang et al. (2017) with these ontogenic genes and marked the BTH-triggered genes as visualized in the heatmaps (R package ComplexHeatmap v.2.15.4) (Gu et al., 2016; Gu, 2022).

### BTH treatment

For *Arabidopsis*, BTH (Actigard 50WG, Syngenta) at a concentration of 50 µM was sprayed onto seedlings. Water was used as a mock control. Samples were collected at 24 h post-treatment.

For cotton, 2-week old plants with one fully expanded true leaf (mature) and one actively expanding (young) true leaf were dipped into the 3-mM suspension of BTH with 0.02% Silwet. Samples were collected in liquid nitrogen at 6 h and 24 h post-dip inoculation, for RNA extraction. This experiment was repeated three times with similar results.

## Data availability statement

The original contributions presented in the study are included in the article/**Supplementary Material**. Further inquiries can be directed to the corresponding authors.

## Author contributions

LH: Writing – original draft, Writing – review & editing, Conceptualization, Data curation, Formal analysis, Investigation, Methodology, Visualization. JM: Writing – original draft, Writing – review & editing, Conceptualization, Data curation, Formal analysis, Investigation, Methodology, Visualization. FK: Data curation, formal analysis, Investigation, Methodology, Visualization, Writing – review & editing. BK: Writing – original draft, Writing – review & editing. LY: Writing – original draft, Writing – review & editing.

## Supplementary material

Supplementary Table 1 Primers used in this study.

Supplementary Table 2 Ontogenic stage preferred gene lists in **Figure 2B**.
